# Factors and Beliefs that Condition the Attitude of Health Science Students towards End of Life in Spain and Bolivia: A Multicenter Study

**DOI:** 10.3390/ijerph17176373

**Published:** 2020-09-01

**Authors:** Sagrario Pérez-de la Cruz, Ivonne Ramírez, César Maldonado

**Affiliations:** 1 Department of Nursing, Physiotherapy and Medicine, University of Almería, La Cañada de San Urbano, 04120 Almería, Spain; 2 Health Science Faculty, Instituto de Investigaciones en Neurodesarrollo, University of San Francisco Xavier de Chuquisaca, Sucre 212, Bolivia; ifrm14@gmail.com; 3 Sagrado Corazón School, Sucre 155, Bolivia; sinchisi@yahoo.com

**Keywords:** students, end of life, training

## Abstract

Health Science students in Spain and Bolivia should be trained in the management of the processes of death and dying of patients. The aim of this study was to examine the degree of training, self-perceived safety and preferences in relation to the care of terminal and non-terminal patients. It was a descriptive, cross-sectional, multicenter study with students of Medicine, Nursing and Physiotherapy in Spain and Bolivia. The following variables were evaluated: care preparation and emotional preparation to caring for terminally ill and non-terminally ill patients, the Death Attitude Profile Revised (PAM-R) and the Bugen Scale for Facing Death. The self-perceived preparation of students for caring for terminally ill patients can be considered “fair” (mean 2.15, SD 0.756), and this was also the case for their perceived emotional preparation (mean 2.19, SD 0.827). In contrast, the score obtained for their preparedness for treating non-terminal patients was higher (mean 2.99 and 3.16, respectively). Working with terminally ill patients, including terminal or geriatric cancer patients, was the least preferred option among future health professionals. The results obtained show a limited preference for end-of-life care and treatment, highlighting a lack of preparation and motivation among health science students in Spain and Bolivia for working with these patients.

## 1. Introduction

The concept of death and dying and our attitudes towards the same have shifted over the last centuries and, even more so, in recent decades. However, the semantics of death have evolved [1]. Advances in medicine and other related sciences now enable the prolongation of life or the maintenance of vital functions beyond previously known limits. Furthermore, the ageing of the population and the consequent increase in people with chronic illnesses means that the number of terminal situations has increased, with a limited life prognosis and intense personal and family suffering, often in a context of health care that is highly technological [2,3].

Professionals trained in the field of healthcare must face death continuously, working in close contact with the family of the person who has just passed away. Professionals not only face fear towards death but also to the process leading up to it, such as enduring the patient’s agony, pain, and loss of physical and mental faculties [4]. The importance of a health professional’s attitude towards the process of death and pain is becoming increasingly recognized [5,6]. Health professionals work in a stressful work environment and witness intense experiences related to the end of life and survival. The quality of their relationship with the patient and the family in the process of death and grieving is key, as well as their ability to manage the moment when they must inform a family of the passing of a family member. Thus, nursing professionals play a major role in this process, typically participating the most in the process of dying. However, in the case of doctors, issues surrounding death are perceived in a different light, as these are supposed to be the best trained professionals in handling the same [7,8]; however, this is not always the case and is often reflected in the lack of direct patient care. Indeed, a relatively small number of studies have shed light on the emotional effects of death on health personnel [5]. Greater exposure to death has been strongly associated with greater work-related stress [9]. In a former study, 61% of the doctors interviewed reported that their most memorable death was still emotionally distressing to recall [10]. Cases that trigger strong emotional reactions include patients with whom the doctor feels an interpersonal connection, patients who were not cured by standard treatment, younger patients, and deaths that lack dignity (e.g., futile resuscitation) [7]. After a patient’s recent death, physicians report feelings of numbness, discomfort when thinking about the patient [7], guilt and stress [11].

For health science students, coping with pain and death is one of the most difficult and stressful tasks they must face [12,13]. Students of medicine and nursing should receive education in the management of the dying process of the patients in their care, making it part of their academic curriculum. Clearly, in order to provide the best possible care for patients in advanced and terminal illness and their families, specific training is needed in aspects of mourning, death, terminal illness and palliative care, both in undergraduate training and during professional performance [13]. However, it is important not to rule out the possibility that the cultural setting, globalization and the progress of technology and medicine influence the ethical values that govern health care and the performance of health professions, understanding the many cultural differences between developed and developing countries [14,15]. A study by Alfred et al. (2013) [16] found that the ethics and professional values of nurses are influenced by the cultures in which they live, learn and practice. These values influence the profession for those students and professionals who go abroad for training and work, influencing their subsequent successful integration into their professional work. According to Barry and Ghebrehiwet (2012) [14], globalization can accelerate cultural development; thus, these authors tested whether it is possible to develop a universal code of ethics for the nursing profession, regardless of culture. Their analysis revealed that cultural values play a fundamental and substantial role in determining the moral conduct of health professionals. Therefore, it is possible to think that health professionals in Latin America may present similar protocols and attitudes to death as those in Spain because they share similar cultural entities, in terms of language, culture, society and religion.

The principal aim of this study was to explore the training and preparation of health science students studying medicine, nursing and physical therapy, in relation to the care of terminal and non-terminal patients and their level of self-confidence in the management of terminally ill patients among a sample of students in Spain and Bolivia.

## 2. Materials and Methods

### 2.1. Participants

A cross-sectional, descriptive, multicenter design was used and approved by the Bioethics Committee of the main author (2016DEC018). This study lasted from November 2018 to June 2019, and during this period of time, data collection and analysis procedures were carried out.

The inclusion criteria for this study were (1) students enrolled in intermediate courses of their degree training, (2) voluntary participation in the study and signing the informed consent, and (3) regular attendance to classes. Those students who were employed in other occupations while completing their university training and those who were not enrolled during the training period stipulated for this study were excluded from this study.

**Figure 1:**
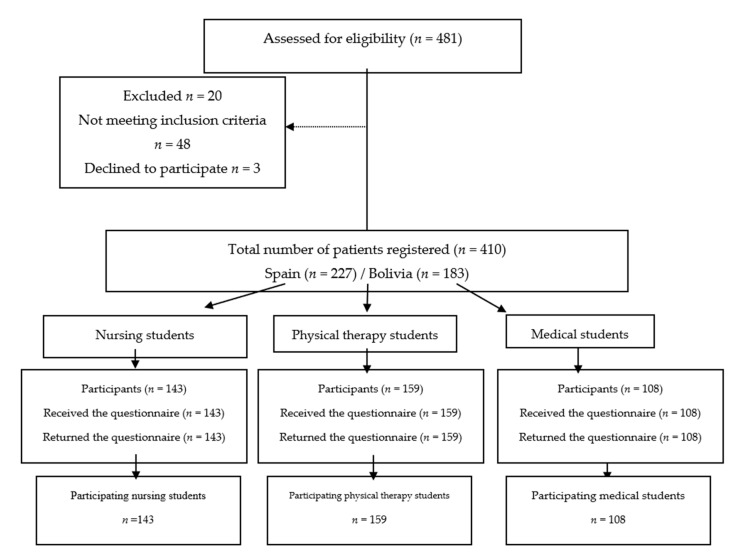
Study design flowchart.

A sample of students from Bolivia and Spain was used (including different faculties, in order to have a diverse sample), to do so, the teachers responsible for the subjects being taught during this academic period were contacted. The sample comprised of 410 students of both sexes, of which 183 were studying medicine, kinesiology/physical therapy and nursing in their second to fourth year at two Bolivian universities, and 227 students were in their second to third year at three Spanish universities. Figure 1 displays the selection process of the study participants.

### 2.2. Outcome Measures

Perceived emotional and technical preparation for the care of terminally ill patients [17]. An ad hoc questionnaire designed for this study was used to ask the participating subjects whether they felt prepared or capable of aspects such as the ability to control pain, also, their communication skills and their ability to treat the array of symptoms that appear during the process of dying. This assessment is a replication of a similar study [17], which was intended to determine students’ predisposition to future work situations. The answers could range from not at all (score of 1), a little (score of 2), to some extent (score of 3), fairly prepared (score of 4) and very prepared (score of 5).

Perceived emotional and technical preparation for the care of patients with general pathology [17]: The aim of these two questions, purposely designed for this study, was to determine whether the perceived emotional and technical preparation was the same when caring for people who are ill but not necessarily at the end of their lives. The scoring system was the same as above.

The Death Attitude Profile Revised (PAM-R) by Wong, Reker and Gesser, revised in 1997, was used to assess attitudes towards death [18]. This scale contains 32 items/statements related to different types of attitudes towards death. This test uses a Likert-type scale, with seven response options ranging from totally disagree (score = 1) to totally agree (score = 7). For each type of attitude, an average scale score is calculated by dividing the total scale score by the number of items on the scale. This instrument consists of five assessed dimensions (attitudes), which are ordered as follows: Component 1, acceptance of closeness (items 4, 8, 13, 15, 16, 22, 25, 27, 28, 31); Component 2, fear of death (items 1, 2, 7, 18, 20, 21); Component 3, death avoidance (items 3, 10, 12, 19, 26); Component 4, escape acceptance (items 5, 9, 11, 23, 29); Component 5, neutral acceptance (items 6, 14, 17, 24, 30).

The Bugen Scale for Facing Death is an instrument that allows us to differentiate between control subjects and subjects in training [19]. Studies such as that by Robbins have confirmed the high internal consistency of the scale (α = 0.89 for *p* < 0.001) for a group of graduate and undergraduate students [19] and (α = 0.90 for *p* < 0.001) in a group of volunteers in palliative care centers [19]. The scale consists of 30 items. Each item is assessed using a Likert-type scale from 1 to 7, with a score of 1 implying total disagreement and 7 total agreement. The final score is obtained by inverting the value of items 13 and 24 and then summing all the scores. This scale measures competence in dealing with death, which is a construct that represents a wide range of human abilities and capacities to deal with death, as well as our beliefs and attitudes towards these capabilities. This scale aims to measure the benefits of valid death education, to monitor whether an educational/training action on death is effective and to emphasize that coping is a desirable outcome of a death education experience.

### 2.3. Study Procedure

In Bolivia, one person was in charge of handing out and collecting the questionnaires, and in Spain, a different person was placed in charge of this, both people were external to the research (so as not to avoid any possible bias). The surveys were completed in the university classrooms and the participants gave their informed consent in the presence of the teacher during class hours.

To allow enough time for students to complete the survey, the teachers responsible for each subject assigned half of their class time (approximately 30 min) in order to inform students about the study and to respond to any questions that arose while students completed the survey. The self-administered, anonymous questionnaires were handed out and collected after 30 min. Enough time was assigned to answer the questions without any time pressures.

### 2.4. Statistical Analysis

The data was entered into IBM^®^ SPSS^®^ Statistics V21.0.0 software (IBM Corp., Armonk, NY, USA). For the analysis, a numerical code was assigned to the data collected, to preserve the anonymity of the participants. Socio-demographic or characterization variables were analyzed. Frequencies, percentages, means and standard deviations were described. In order to compare the scores obtained between the countries and to conclude whether such differences were statistically significant, parametric tests were used, based on the normal distribution of data. To compare the scores obtained between countries, the Student’s T-test for independent samples was used. A value of *p* < 0.05 was considered statistically significant.

## 3. Results

Of the 410 students who participated in the study, 227 were students from Spanish universities (55.3% of the total sample), whereas 183 were from universities in Bolivia (44.6%). The demographic characteristics of the sample are shown in Table 1.

Table 2 displays the descriptive and comparative analysis of the scores regarding the perceptions of students in Spain and Bolivia regarding their ability to face the care of terminal and non-terminal patients. Statistically significant differences (*p* < 0.001) were found in the results shown in terms of knowing how to face the care of terminally ill and non-terminally ill patients, as well as their ability to emotionally cope with facing the care of patients who are close to the end of their lives. For the item of emotional preparation for the care of non-terminally ill patients, no differences were observed, with students overall displaying confidence in their ability to fulfill this task in their daily work. The results indicate that students in Bolivia feel better prepared than Spanish students for the care of terminally ill and non-terminally ill patients, as well as feeling emotionally prepared for the care of terminally ill patients. However, both samples are emotionally prepared to care for patients with non-terminal illnesses.

The descriptive and comparative scores of the items regarding Death Attitude Profile Revised (PAM-R) for both groups are featured in Table 3. According to the components of this scale, it is important to show the items that conform component 3 (avoidance of death), in which no statistically significant differences were found in the results of both samples, whereas for component 1 (acceptance of rapprochement) statistically significant findings were observed in all the values evaluated. Regarding the remaining components (fear of death, escape acceptance and neutral acceptance), a diversity of responses were observed, with the highest number of statistically significant responses found for neutral acceptance, in three of the five items, fear of death in four out of six items; whereas in escape acceptance statistically significant findings were found in only two of the values evaluated.

Table 4 shows the results obtained in the Bugen Scale for Facing Death. This scale measures competence in facing death and also our beliefs and attitudes towards these abilities. Health science students of both countries revealed a lack of confidence in their competence when facing death and feeling prepared to face these stressful situations, which will no doubt arise when fulfilling their work duties.

## 4. Discussion

Currently, death is a taboo subject in Western culture, unlike other cultures, where death is perceived as a natural part of life. In these matters, we are sold the omnipotence of technical and technological means in the field of medicine and health in general, generating insecurity, discomfort and anxiety when a terminal illness develops leading to death [20,21,22]. Moreover, the professional’s attitude towards death influences their response to the requests of the dying person’s family [21,23]. A study by Benbunan-Bentata et al. (2007) [24] also highlights a further aspect that is very important during this transition between life and death, concerning the presence, involvement, affection and effects of the emotions shown by the health professionals (or, in the case of this study, in future health professionals) and how they face the anxiety and stress derived from this situation. These feelings are sometimes described with words such as “unpleasant”, “bad”, “painful”, “heartbroken”, “frustrated”, “terrible”, “horrible”, “tragic”, “dramatic”, “distressing”, “hard”, “not knowing how to act”, and “shocking”, as if the person were describing their own death. These results are in line with numerous studies evaluating anxiety and fear of death [21,22,25], highlighting feelings of helplessness and insecurity.

To successfully overcome this stressful situation, it is necessary to carry out an assessment program, including communication and information skills, psychosocial support, as well as assigning importance to bioethical issues and interdisciplinary work among professionals, and specifically among doctors and nurses as they are closer to situations of death and dying. Students and professionals should not only be taught technical and scientific knowledge, but they should also know how to behave as individuals who are sensitive to human suffering. That is why the health-patient relationship is defined as an essentially humanistic relationship. It is necessary to encourage an environment in which the values, customs and beliefs of the individual are respected, helping the patient to maintain, develop or acquire personal autonomy, with respect and determination towards the person’s decision, guided by professional criteria, in the patient’s own interest [26].

An attitude of empathy on behalf of professionals towards suffering is essential for accompaniment at the end of life. Likewise, the individualization of the interventions and the recognition of interpersonal emotional variability in the experience of death constitutes the framework of end-of-life care [17].

There are few publications on this subject in Latin America. However, it is worth highlighting some studies on this subject, which analyze the attitude of certain groups of the population towards the proximity of death. Motta et al. (2016) [27] conducted a study on beliefs, attitudes and anxiety surrounding death in a multidisciplinary team of oncological palliative care professionals. The main attitudes of health staff towards the terminal patient and death include feelings of greater responsibility, concerning care for life and promotion of personal growth to accept their own death. This study concludes that it is important to reflect on death and not to avoid doing so, not only because of age or the evolution of the human being. Gala et al. (2002) [8] conducted a study on psychological attitudes towards death and grieving, showing that anxiety in the face of death is also very closely related to personal and cultural history and styles of coping with separation and change. A series of affective and emotional components predominate in the attitudes of health professionals in the face of death. Thus, access to appropriately structured and accurate information and experience is required to modify the initial attitudes towards death and the suffering of patients [6,28].

Other psychological studies carried out in Latin America have examined the effects of stress among doctors and medical students [29], based on the concept of patient death with dignity, with both parties emphasizing the importance of humanism in good health practices. These results are in line and with previous studies among medical students in Spain [30].

Theoretically, if students do not feel emotionally prepared, they will be less able to care for terminally ill and non-terminally ill people, as demonstrated in research study by Colell Brunet et al. (2010) [17], who obtained higher scores in emotional preparation than technical care, finding greater difficulties in interrelationship and communication with terminally ill patients, as well as with their close family environment [31]. This is in line with the results obtained in our samples from Spain and Bolivia, without distinguishing among professionals, since in our study, emotional preparation was also superior to the perceived preparation for technical care. This similarity may be due similarities in the undergraduate training, in the initial courses, or to personal experiences and/or clinical practices.

Moreover, it is also worth noting the low score obtained in terms of the perceived academic and technical preparation for caring for terminally ill patients, as found in other similar studies [32,33]. Moreover, certain subjects are not taught on a mandatory basis; thus, palliative care is an optional subject, which is not typically sought-after by students, who consider that work with this type of patient is “very sad and depressing”. Students do not consider that they need specific preparation to care for these patients as they are different to other hospitalized patients [3]. In part, this conception stems from the university centers themselves, which should go deeper into showing the reality that students are going to face in real life, either because the processes of illness usually take place in a final stage of life, because life expectancy is high, or to make them aware that our users will be people for whom a way out of their condition may be death and not always total and full recovery/rehabilitation.

The need to improve the training of future professionals [34], and licensed professionals also, in matters related to terminal illness (most common in advanced chronic and degenerative diseases) is a necessary objective for health administrations. However, attention to personal factors concerning the professionals who work with these patients and their families is still ignored or avoided, both at the care level and in proposals for improvement and accompaniment.

Therefore, it follows that students and professionals who participate in well-designed training programs improve their coping and thus feel more confident and prepared when working with terminally ill and dying patients. These professionals will be better prepared and qualified when working in palliative care services or in those situations where they must care for patients in end-of-life processes.

One of the main limitations of the present study is related to the risk of bias in the selection of the sample, as this was based on a group volunteers who fulfilled the inclusion criteria, and there was a lack of randomization. Moreover, it would be interesting to conduct the same study on health science students who have already experienced more continuous contact with patients so that they can express their opinion in a way that is more in line with their lived experience. Future studies should be conducted to further explore this subject and identify the training needs of health science students in the topic of end of life and patient death, together with larger samples to allow us to extrapolate the results obtained. Likewise, it would be appropriate to present the study findings to the different faculties of health sciences of both countries so that they understand and value the need to implement and develop their curriculum in this matter.

## 5. Conclusions

Students tend to reject end-of-life care and treatment, believing that preparation for caring for this type of patient should begin during undergraduate training, since changes in behavior and motivation towards this field of work must begin early (adequate design of the study curriculum), requiring time to fully acquire these skills.

This study also highlights a similarity in the perceptions regarding the end of life of students of Health Sciences in Spain and Bolivia. This is not a very attractive field of work for students, who perceived a lack of preparation to face the end of their patients’ lives. However, we found that students in Bolivia were slightly more open and particularly more so among nursing students, compared to medicine and physical therapy students.

## Figures and Tables

**Table 1:** Distribution of students according to their degree and gender.

Degree	Total (%)	Men (%)	Women (%)
Nursing	143(35.03)	30 (20.8)	113 (79.2)
Physical therapy	159 (38.7)	81 (59.9)	78 (49.1)
Medicine	108 (26.27)	21 (19.4)	87 (80.6)

**Table 2:** Perceived emotional preparation and ability to care for terminal and non-terminal patients.

Sections	Country		Difference Means	Student’s *t*-Test	
	Spain	Bolivia		t(546)	*p*-Value
Care for terminal patients	2.27 (0.76)	3.13 (0.8)	−0.86	−12.797	<0.001
Care for non-terminal patients	2.98 (0.61)	3.33 (0.8)	−0.35	−5.973	<0.001
Emotional preparedness terminal patients	2.23 (0.85)	3.18 (0.93)	−0.95	−12.391	<0.001
Emotional preparedness non-terminal patients	3.12 (0.68)	3.33 (0.8)	−0.21	−3.377	0.001

**Table 3:** Descriptive and comparative items of the PAM-R scale, by country.

PAM-R Items	Country		Difference Means	Student’s *t*-Test	
	Spain	Bolivia		t(546)	*p*-Value
COMPONENT 1: Acceptance of closeness	26.61 (13.16)	45.98 (13.18)	0.02	0.59	0.404
COMPONENT 2: Fear of death	25.93 (9.89)	27.03 (9.15)	0.74	2.22	0.137
COMPONENT 3: Death avoidance	19.06 (8.60)	19.08 (7.88)	0.02	2.97	0.085
COMPONENT 4: Escape acceptance	13.13 (6.70)	19.47 (8.50)	6.34	7.79	0.005
COMPONENT 5: Neutral acceptance	26.98 (5.43)	26.58 (6.12)	0.40	5.11	0.024

**Table 4:** Descriptive and comparative analysis of items on the Bugen’s scale by country.

Bugen’s Scale Items	Country		Difference Means	Student’s *t*-Test	
	Spain	Bolivia		*t*(546)	*p*-Value
BS1	3.22 (1.64)	4.19 (1.73)	−0.97	−5.482	<0.001
BS2	3.09 (1.08)	3.81 (1.7)	−0.72	−4.281	<0.001
BS3	4.42 (1.66)	3.98 (1.91)	0.44	3.859	<0.001
BS4	4.52 (1.22)	3.77 (2.27)	0.75	3.366	0.001
BS5	3.59 (1.37)	3.91 (2.03)	−0.32	−0.627	0.426
BS6	3.44 (1.31)	3.51 (1.81)	−0.07	−0.429	0.677
BS7	5.22 (1.08)	4.63 (1.77)	0.59	2.384	0.012
BS8	3.14 (1.63)	3.57 (2.01)	−0.43	−2.426	0.007
BS9	4.03 (1.39)	3.58 (1.69)	0.45	2.229	0.034
BS10	3.42 (1.53)	4.25 (1.94)	−0.83	−4.672	<0.001
BS11	5.19 (1.42)	4.59 (1.76)	0.6	3.415	0.001
BS12	4.85 (1.66)	4.34 (1.53)	0.51	2.022	0.041

BS 1, I feel prepared to face my dying process. BS 2, I feel prepared to face my death. BS3, I can express my fears about dying. BS 4, I can talk about my death with family and friends. BS 5, I will be able to cope with future losses. BS 6, I feel able to handle the death of other close to me. BS 7, I know how to listen to others, including the terminally ill. BS 8, I know how to speak to children about death. BS 9, I am able to spend time with the dying if I need to. BS 10, I can help someone with their thoughts and feelings about death and dying. BS 11, I would be able to talk to a friend of family member about their death. BS 12, I can communicate with the dying.
